# Network Modeling for Functional Magnetic Resonance Imaging (fMRI) Signals during Ultra-Fast Speech Comprehension in Late-Blind Listeners

**DOI:** 10.1371/journal.pone.0132196

**Published:** 2015-07-06

**Authors:** Susanne Dietrich, Ingo Hertrich, Hermann Ackermann

**Affiliations:** Department of General Neurology, Hertie Institute for Clinical Brain Research, Center for Neurology, University of Tübingen, Hoppe-Seyler-Str. 3, D-72076 Tübingen, Germany; CEA.DSV.I2BM.NeuroSpin, FRANCE

## Abstract

In many functional magnetic resonance imaging (fMRI) studies blind humans were found to show cross-modal reorganization engaging the visual system in non-visual tasks. For example, blind people can manage to understand (synthetic) spoken language at very high speaking rates up to ca. 20 syllables/s (syl/s). FMRI data showed that hemodynamic activation within right-hemispheric primary visual cortex (V1), bilateral pulvinar (Pv), and left-hemispheric supplementary motor area (pre-SMA) covaried with their capability of ultra-fast speech (16 syllables/s) comprehension. It has been suggested that right V1 plays an important role with respect to the perception of ultra-fast speech features, particularly the detection of syllable onsets. Furthermore, left pre-SMA seems to be an interface between these syllabic representations and the frontal speech processing and working memory network. So far, little is known about the networks linking V1 to Pv, auditory cortex (A1), and (mesio-) frontal areas. Dynamic causal modeling (DCM) was applied to investigate (i) the input structure from A1 and Pv toward right V1 and (ii) output from right V1 and A1 to left pre-SMA. As concerns the input Pv was significantly connected to V1, in addition to A1, in blind participants, but not in sighted controls. Regarding the output V1 was significantly connected to pre-SMA in blind individuals, and the strength of V1-SMA connectivity correlated with the performance of ultra-fast speech comprehension. By contrast, in sighted controls, not understanding ultra-fast speech, pre-SMA did neither receive input from A1 nor V1. Taken together, right V1 might facilitate the “parsing” of the ultra-fast speech stream in blind subjects by receiving subcortical auditory input via the Pv (= secondary visual pathway) and transmitting this information toward contralateral pre-SMA.

## Introduction

Vision loss may allow for cross-modal reorganization processes within the visual system in association with distinct perceptual exigencies. Many functional imaging studies indicate the central-visual system to contribute to the enhanced processing of nonvisual stimuli in blind subjects. For example, striate cortex shows significant hemodynamic activation during Braille reading [1–4], auditory motion detection [5], syntactic and semantic speech processing [6] as well as cognitive language tasks such as verb generation, production of mental images based upon animal names, and retrieval of verbal-episodic memory contents [7–9]. Furthermore, in some analogy to the fast-reading skills of sighted subjects, blind people can manage–by repeated exposure to accelerated verbal utterances using, for example, computer screen readers–to understand spoken language at enhanced speaking rates of up to 22 syllables per second (syl/s)–an accomplishment exceeding by far the limits of untrained subjects (ca. 8 syl/s). A previous functional magnetic resonance imaging (fMRI) investigation [10] found in a late-blind subject group that hemodynamic activity in right-hemispheric primary visual cortex (V1), bilateral pulvinar (Pv), and the anterior part of the left-hemispheric supplementary motor area (pre-SMA)–in addition to the “classical” perisylvian language zones (left-hemispheric inferior frontal gyrus, bilateral superior/middle temporal gyrus/sulcus)–covaried with the capabilities of ultra-fast speech (16 syl/s) comprehension. Based upon fMRI studies [10–13] as well as investigations using magnetencephalography (MEG) [14], it has been suggested that right-hemispheric V1 plays an important role with respect to the (i) perception of ultra-fast speech features, i.e. syllable onsets, and (ii) forwarding of syllabic information via left-hemispheric pre-SMA to the speech processing structures, i.e. inferior frontal gyrus. Furthermore, Pv has been assumed to synchronize–driven by acoustic input–striate cortex with the central-auditory system during ultra-fast speech perception [10, 12], based upon cross-modal subcortical pathways that in sighted individuals subserve audiovisual coincidence detection [15] and the control of visual attention [16].

As an extension of our preceding fMRI group study [10], the present investigation addresses network modeling (dynamic causal modeling) regarding brain regions the activation of which had shown covariance with behavioral performance in ultra-fast speech comprehension. Considering the hypothesized mechanism [12], connectivity analysis will focus on a single network comprising the right-hemispheric primary auditory cortex (A1), ipsilateral Pv, the right-hemispheric V1, and left-hemispheric pre-SMA. Based on anatomical and functional considerations, Pv, A1, and V1 interaction may be considered as a perceptual input structure while A1, V1, and pre-SMA coupling refers to output from sensory regions toward frontal cortex:

### Input sub-network (Fig 1)

**Fig 1:**
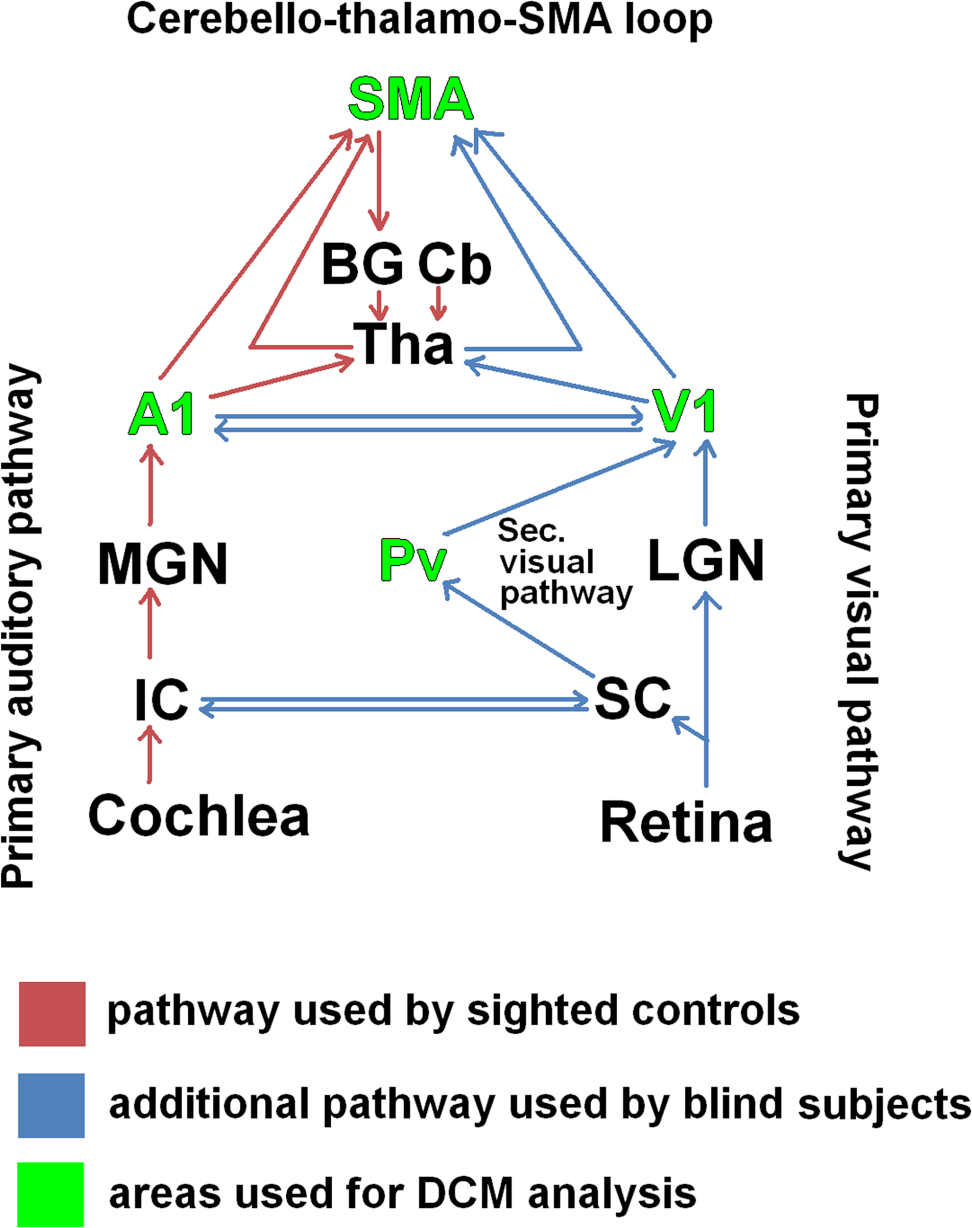
The hypothesized network. An anatomically and functionally based model describing the in- and output stream of the right primary visual area (V1): Besides the primary auditory pathway (red arrows), auditory information coming from the cochlear thread at the level of the tectum, inferior and superior colliculus (IC, SC) (an audiovisual interface), and the secondary visual pathway, pulvinar (Pv), into V1 (blue arrows). Further, sensory areas, A1 and V1, modulate via the thalamus (Tha) the supplementary motor area (pre-SMA) in order to optimize temporal processing and predictive coding (cerebello-thalamic-pre-SMA loop). Investigating this network, four areas (green) were used for the DCM analyses. BG = basal ganglia, Cb = cerebellum, LGN = lateral geniculate nucleus, MGN = medial geniculate nucleus.

Considering Pv, i.e., the audiovisual subcortical interface, tract-tracing studies in monkeys found both the ascending auditory pathways as well as the optic tracts to send convergent collateral fiber tracts to deep layers of the superior colliculus (SC), and the respective target neurons, in turn, project via Pv to auditory as well as visual cortex [17]. Thus, the visual pathway consists of two distinct streams: (i) the primary visual pathway extending from retina via lateral geniculate nucleus (LGN) to V1, transferring retinotopic visual information and (ii) the secondary visual pathway (ca. 10% of the retino-geniculate fibers) passing SC and Pv before reaching visual cortex (V1 as well as secondary visual areas). The latter has been identified as a non-image forming visual stream [18]. It has been mentioned in the context of very fast (backward-masked with regard to conscious perception) visual processing such as the perception of affective faces [19–20]. Regarding ascending auditory information, neural responses coming from the cochlea reach the inferior colliculus (IC) in the midbrain–after passing the auditory brainstem nuclei–and feed via the medial geniculate nucleus (MGN) into A1. Thereby, IC was found to be additionally connected with SC providing the possibility of audiovisual interactions [21–22]. In consideration of this network architecture, the secondary visual pathway is hypothesized to provide the central-visual system with auditory input. Given, furthermore, direct anatomical connections between auditory and visual areas [23–25], early multisensory convergence processes at the cortical level must be assumed as well, as demonstrated, e.g., by means of transcranial magnetic stimulation [26]. Considering our hypothesis about the V1 input network, coupling between Pv and V1 is expected in blind subjects, indicating recruitment of the secondary visual pathway (Fig 1). However, in addition to Pv-V1 connectivity, coupling between A1 and V1 may also be expected. By contrast, in sighted controls V1 may not to be connected to auditory input during unimodal listening since activation of audiovisual pathways might be bound to the presence of coinciding visual input.

### Output sub-network (Fig 1)

It has been suggested that speech processing in case of continuous listening to sentence materials relies on a subcortico-cortical multifunctional network, coordinating prosodic timing with the left-frontal speech and language processing network, including mechanisms of predictive coding of events to optimize speech perception [27–29]. The latter aspect has been described as a loop including the cerebellum (Cb), thalamus (Tha), pre-SMA, and basal ganglia (BG) [28]. In case of acoustically cued simple motor tasks, SMA was found to receive input from auditory cortex, as suggested by a study using Granger causality as a measure of connectivity [30]. Applying the concept to ultra-fast speech perception in blindindividuals, right V1 seems to be recruited in order to support right A1 with respect to encoding and forwarding of ultra-fast speech features and, thus, V1 is also expected to modulate the cerebello-thalamo-pre-SMA loop. As concerns our hypothesis about the V1 output network in blind subjects performing ultra-fast speech comprehension, coupling between V1 and pre-SMA is expected, in addition to the normal A1-to-pre-SMA connectivity that can be presumed for sighted controls (Fig 1).

In order to test these hypotheses, dynamic causal modeling (DCM)–a generic approach for inferring unobserved neuronal states from measured brain activity and, thereby, calculating connectivity patterns between activated areas [31]–was applied to the fMRI data of Dietrich and colleagues [10].

## Methods

### Participants

A total of eleven late-blind (9 males; mean age = 37.9 years, *SD* = ±13.05; blindness onset after the age of 7 years) and eleven normal sighted subjects (7 males; mean age = 30.8 years, *SD* = ±9.56) participated in the DCM analysis (Table 1). In all instances, a peripheral origin of blindness could be established, but the participants represented a rather heterogeneous group with respect to their age at the onset of vision loss. Furthermore, some of the blind subjects had minor residual visual capabilities such as light sensitivity, but all of them showed no or–if at all–highly diminished visual acuity. Visual acuity was assessed by a pattern discrimination task (recognition of two points with minimal distance) measured as test distance (m) divided by minutes of arc (ICD-9-CM, International Classification of Diseases, Ninths Revision, Clinical Modification, http://www.cdc.gov/nchs/icd/icd9cm.htm). The present group of late-blind participants comprised nine subjects with visual acuity of 0.01 or less (almost total loss of vision) and two subjects with profound vision loss (visual acuity = 0.02, 0.03).

**Table 1:** Clinical and behavioral data of the vision-impaired and normally sighted subjects.

Subjects	Performance of speech perception (%)		Age of blindness onset	Etiology of blindness	Characterization visual deficits, in parentheses visual acuity *
	Ultra-fast utterances	Moderately fast utterances			
B01	93	94	7	retinal detachment	no residual visual perception (0)
B02	91	85	14	retinal pigmentosa	no statement
B03	72	80	26	macular degeneration	light perception (0.01)
B04	67	85	7	hereditary vision loss (gene mutation)	no residual visual perception (0)
B05	65	89	44	retinitis pigmentosa	light perception (0.01)
B06	64	89	37	glaucoma	no residual visual perception (0)
B07	62	82	24	retinal damages	no residual visual perception (0)
B08	60	84	17	uveitis intermedia	residual visual perception (0.03)
B09	57	99	13	retinal detachment	no residual visual perception (0)
B10	39	74	18	retinal damages	no residual visual perception (0)
B11	0	65	47	eye cataract, glaucoma	residual vision strongly reduced (0.02)
S01	16	85	-	control	
S02	16	99	-	control	
S03	11	69	-	control	
S04	6	61	-	control	
S05	5	89	-	control	
S06	4	71	-	control	
S07	9	81	-	control	
S08	7	85	-	control	
S09	16	96	-	control	
S10	6	88	-	control	
S11	8	78	-	control	

* Visual acuity measures optic resolution in units of 1/minutes of arc, assessed at a distance of 1m. Values below 0.01 were listed as zero. Abbreviations: B = late-blind, S = normally sighted.

### Behavioral repetition task

In order to obtain a quantitative behavioral measure of an individual’s capability to understand ultra-fast speech utterances, each subject performed a sentence repetition task based upon sentence utterances produced at an ultra-fast and moderately fast speaking rate (8 and 16 syl/s; screen reader software JAWS 2008, male voice, “eloquence” formant synthesizer, http://www.freedomsci.de, see [10]). The test materials (see [10] for audio-examples) were presented to the participants via loudspeakers within a sound-attenuated room. Subjects were asked to repeat them “as good as possible”, even when they had failed to “grasp” all the words of a stimulus. The spoken repetitions were digitally recorded and underwent subsequent assessment in terms of the determination of the percentage of correctly reproduced words at each rate condition (lexical items, irrespective of minor grammatical errors such as deviant singular or plural endings). Comprehension of ultra-fast speech utterances (16 syl/s)–in terms of the percentage of correctly reproduced items of 10-word sentences outside the scanner–extended in blind listeners from 0 to 93%–a wide range of performance allowing for subsequent correlation analyses (Table 1). In sighted individuals, performance level consistently fell below 16% (Table 1). All of them were right-handed (Edinburgh handedness inventory) native German speakers without a history of neurological problems or hearing deficits (determined by means of an audiogram). The study design had been approved by the ethics committee of the University of Tübingen, and written informed consent was obtained prior to the MRI measurements from all subjects. For detailed characterization of the behavioral testing see Dietrich and colleagues [10].

### Stimuli

The test materials encompassed 40 different text passages (sentences) transformed into acoustic speech signals by means of a formant synthesizer (screen reader software JAWS 2008; male voice; http://www.freedomsci.de). All utterances were first recorded at a normal speaking rate (4–6 syl/s). Using the speech processing software Praat (version 4.5; http://www.fon.hum.uva.nl/praat/), 20 out of the total of 40 sentences were compressed to a moderately fast (8 syl/s) and the remaining 20 items to an ultra-fast syllabic rate (16 syl/s). In addition, both subsets of the test materials were stored as time-reversed speech signals (backward played sentences), serving as spectrally matched, but unintelligible control items to the two forward-conditions. For a detailed characterization of stimuli see Dietrich and colleagues [10].

### FMRI data acquisition

All functional imaging sessions included two repetitions of the 20 stimuli of each type (fw8, fw16, bw8, bw16), altogether 160 stimuli as well as 40 silent baseline intervals (scanner noise). The test materials were subdivided into five runs and presented in randomized order (event-related design) at an inter-stimulus interval of 9.6 s (jitter = ± 1.4 s, steps of 0.2 s) via headphones (for detailed characterization of data acquisition see [10]). Prior to scanning, participants were instructed to listen carefully to the applied auditory stimuli and to try to understand the displayed verbal utterances. The experiment was run on a 3 Tesla MRI system (Magnetom TRIO; Siemens, Erlangen, Germany), using an echo-planar imaging sequence (echo-time = 30 ms, 64 × 64 matrix with a resolution of 3 × 3 mm^2^, 27 axial slices across the whole brain volume, TR = 1.6 s, slice thickness = 4 mm, flip angle = 90°, 270 scans per run). The scanner generated a constant background noise throughout fMRI measurements, serving as the baseline condition of the experimental design (null event). Anatomical images required for the localization of the hemodynamic responses were obtained by means of a GRAPPA sequence (T1-weighted images, TR = 2.3 s, TE = 2.92 ms, flip angle = 8°, slice thickness = 1 mm, resolution = 1 × 1 mm^2^) of a bi-commissural (AC-PC) orientation.

### Data analysis

Preprocessing of the data encompassed slice time and motion correction, normalization to the Montreal Neurological Institute (MNI) template space, and smoothing by means of an 8 mm full-width half maximum Gaussian kernel (SPM5 software package; http://www.fil.ion.ucl.ac.uk/spm). For the sake of statistical analysis, the blood oxygen level-dependent (BOLD) responses were modeled by means of a prototypical hemodynamic function within the context of a general linear model (event durations = 4 s). Any low-frequency temporal drifts were removed using a 128 s high-pass filter.

The evaluation of the functional imaging data was reported in Dietrich and colleagues [10], comprising (i) the hemodynamic effects of the various stimulus categories (fw8, fw16, bw8, bw16 versus baseline) computed separately for blind and sighted individuals, (ii) differences between blind and sighted groups under the various conditions and (iii) covariance analysis of hemodynamic responses with behavioral performance of ultra-fast speech comprehension. Considering the latter analysis, significant covariance of the capability of understanding ultra-fast speech with hemodynamic activation under the ultra-fast speech condition (forward 16 syl/s versus null event, across blind and sighted individuals) emerged, among others, within right-hemispheric V1 (x, y, z, 15, -102, 6), left-hemispheric pre-SMA (x, y, z, -6, 9, 60), and right-hemispheric Pv (x, y, z, 18, -30, -6). Thus, the specification of the model space for the present DCM analysis was based, among others, on these group coordinates. Thereby, only blind subjects showed significant Pv and V1 activation (correlating with their capacity of ultra-fast speech comprehension), whereas sighted individuals–not performing ultra-fast speech comprehension–did not activate Pv and V1 during speech perception. As concerns the present DCM analysis, an additional design matrix using the general linear model was calculated for each subject (SPM8, software package; http://www.fil.ion.ucl.ac.uk/spm) in terms of specifying the following four speech conditions and excluding the null-event (baseline condition): fw8, fw16, bw8, bw16. At the second level, two-sample *t* tests were performed on this DCM design matrix analyzing a conjunction over both subgroups, blind and sighted individuals, across all four conditions. In order to obtain a coordinate within right A1 (x, y, z, 51, -12, 3) for the DCM analysis, conjunction analysis ensured that both groups showed significant BOLD responses at the identified peak and, thus, time series extraction from identical regions entered the DCM analysis. This approach avoids a bias towards any subgroup or experimental condition with respect to the estimated parameters. Regarding this contrast, a family wise error (FWE) corrected threshold of *p* = 0.05 was applied including, additionally, a mask of right A1 on the basis of cytoarchitectonic probability maps (SPM anatomy toolbox, [32]).

### Dynamic causal modeling

Dynamic causal modeling (DCM, see [31]) was applied to delineate neural coupling describing the in- and output interactions of right V1 in blind subjects performing ultra-fast speech comprehension in contrast to low-performing sighted subjects. Thereby, DCM is an approach for inferring unobserved neural states from measured hemodynamic activation. Based on a bilinear model of neural population dynamics combined with a hemodynamic forward model describing the transformation of neural activity into a BOLD signal [31], three parameters are estimated: (i) direct influence of a stimulus on regional activity (= driving input), (ii) interregional influence in the absence of experimental modulation (= intrinsic connectivity), and (iii) modulatory influence to the intrinsic coupling (= modulatory input). In the present investigation, only the first two parameter sets (driving input and intrinsic connectivity) were applied. Furthermore, family-wise Bayesian model selection (BMS), a method to select a family of models with the highest evidence with respect to connectivity patterns, was applied in combination with Bayesian model averaging (BMA) within families, an approach to make inferences about different subject groups (sighted, blind) who are hypothesized to use different families/models [33].

#### Time series extraction

**Fig 2:**
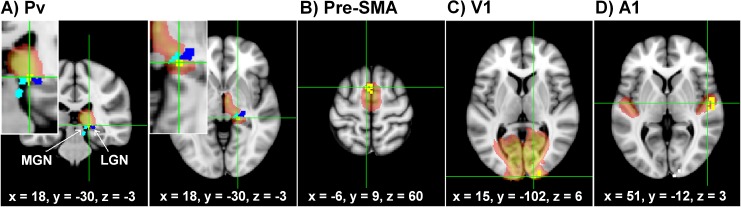
Volumes of interest used for the model space definition. Location of the domain in which blind subjects showed their individual peak coordinates (yellow) within cytoarchitectonic masks of (A) pulvinar (Pv), (B) supplementary motor area (pre-SMA), (C) primary visual area (V1), and (D) primary auditory area (A1). Note the Pv peaks did neither overlap with MGN (light blue) nor lateral geniculate nucleus (LGN) (dark blue). All selected structures overlaid on a T1 template.

FMRI time series were extracted from activation peaks in the above described four regions (right A1, Pv, V1, and left pre-SMA see Fig 2). Because local maxima of activation differ across subjects, time series extraction relied on individually adjusted coordinates by using a combination of functional and anatomical constraints: (i) an individual significance level of *p* < 0.05 uncorrected, (ii) maximum distance from group peak (see covariance analysis of [10]) of 4 mm Pv, and 8 mm for A1, V1, pre-SMA), (iii) location within the respective cytoarchitectonically defined mask (A1, Pv, V1, pre-SMA; SPM anatomy toolbox, [32]). If a participant did not show significant BOLD responses in a search region, e.g. in case of V1 in sighted controls, group coordinates (resulting from the covariance analysis [10]) were taken. S1 Table lists the coordinates of individual peaks and also indicates whether a subject did not show significant BOLD responses in a region. After identifying these peaks, a sphere around each peak (4 mm radius for Pv, 8 mm radius for A1, V1, pre-SMA) was defined as the respective volume of interest.

#### Model specification

**Fig 3:**
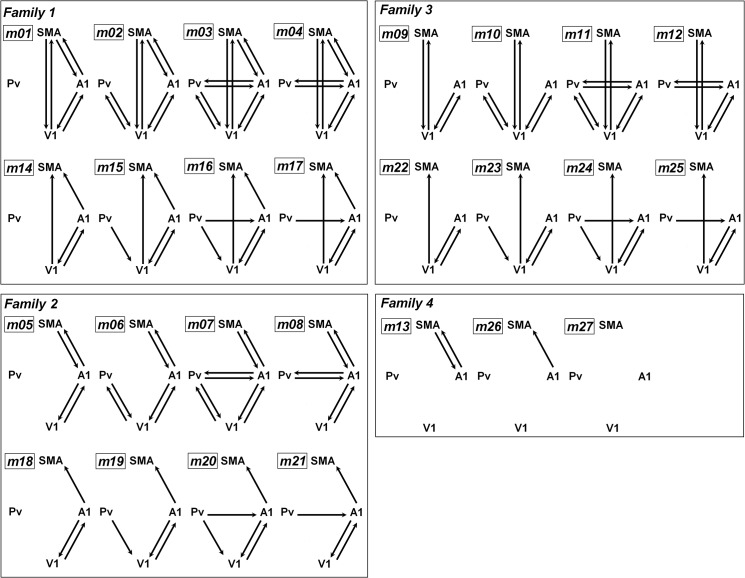
Model specification. Model comparison was performed on family-level inferences: Models were subgrouped into four families specified by (Family 1) coupling between V1/A1 and SMA, (Family 2) coupling between A1 and SMA, (Family 3) coupling between V1 and SMA, and (Family 4) those with/without coupling between A1 and SMA, but without any connection to/from Pv/V1. Within the families, models consisted of either (i) bidirectional, (ii) absent, or (iii) unidirectional connectivity regarding the Pv/V1 coupling to/from the remaining areas. Furthermore, unidirectional models were configured hierarchically in the way that Pv projected forward to A1/V1 which again projected forward to SMA. Backward projections were only defined with respect to bidirectional models, not in an unidirectional way. A priori, driving input on Pv/A1, bidirectional A1-V1, and absent Pv-pre-SMA connectivity was assumed.

Hypothesizing the recruitment of the secondary visual pathway (see Fig 1), A1 and Pv were considered as driving input regions, whereas pre-SMA was modeled as an area with higher cortical functions. Both input regions (A1, Pv) were specified to be driven by forward and backward moderately speech (both speech rates: 8 and 16 syl/s). Connections between A1 and V1 were assumed to be bidirectional, but no direct Pv-pre-SMA connections were included (peak location within Pv was found to be within the inferior lateral region and, thus, hypothesized to interact with sensory rather than frontal cortex). The resulting models were assigned to four “families” as shown in Fig 3 (Fam. 1: coupling between V1/A1 and SMA; Fam. 2: coupling between A1 and SMA; Fam. 3: coupling between V1 and SMA; Fam. 4: no connections to/from Pv and V1). Models of each family consisted of either bidirectional, absent, or unidirectional connectivity regarding the Pv/V1 coupling to/from the remaining areas. Furthermore, unidirectional models were configured hierarchically in the way that Pv (subcortical) projected forward to A1/V1 (sensory cortex) which again projected forward to SMA (higher cognitive area). Backward projections were only defined for bidirectional, but not in case of unidirectional connections (Fig 3).

#### Inference on model space (Bayesian model selection)

Bayesian model selection (BMS) as implemented in SPM8 [33–34] was applied to identify the most likely family and included blind as well as sighted subjects. BMS results report the family posterior means as well as exceedance probability which correspond to the belief that one family is more likely than any other. The posterior means were computed from samples drawn from densities describing the distribution over the probability of how frequent a family of models in the population is. Random effects analyses for BMS were used, because high inter-subject variability was assumed, first, due to the high-level cognitive task and, second, to the inclusion of blind and sighted [34–36].

#### Inference on model parameters (Bayesian model averaging)

After identification of the optimal family, Bayesian model averaging (BMA) was applied, to choose the mean and standard deviation of DCM parameters for each subject. This approach computes weighted averages of each model parameter, where the weighting is given by the posterior probability for each family. Separately for blind and sighted subjects, parameter estimates of the driving input and intrinsic connectivity were tested for significance against zero (= no connectivity or no input) using two-tailed one-sample *t* tests (IBM SPSS Statistics 20). Significant values were corrected (Bonferroni Holm) for eight comparisons regarding the driving input (two areas: Pv/A, four conditions: fw8, bw8, fw16, bw16) as well as ten comparisons regarding intrinsic connectivity (five connections modulated to forward or backward direction). Further, correlation analysis was performed between parameters and (i) behavioral performance of ultra-fast speech comprehension (percent correctly reproduced words during the repetition task) as well as (ii) the age of blindness onset. Although values of behavioral performance and blindness onset did not significantly differ from normal distributions (Kolmogorov-Smirnov test), rank correlation analysis was performed (Spearman Rho) in order to obtain more robust inferences, in the view of the small sample size, the presence of outliers, and the rather skewed distribution of behavioral performance. On the basis of our hypotheses (see Fig 1), significant correlations of behavioral performance was expected with connectivity from Pv to V1 and from V1 to pre-SMA, since these areas were found to covary significantly with the capability of understanding ultra-fast speech [10]. Thus, Bonferroni Holm correction was not applied to correlational analyses regarding the performance. By contrast, since correlations with the age of blindness onset were not hypothesized, Bonferroni Holm correction was applied to this external parameter. Furthermore, significant differences of parameter estimates (connections strength and driving input) between blind and sighted subjects were tested by means of a one way ANOVA.

## Results

### Model selection (BMS)

**Fig 4:**
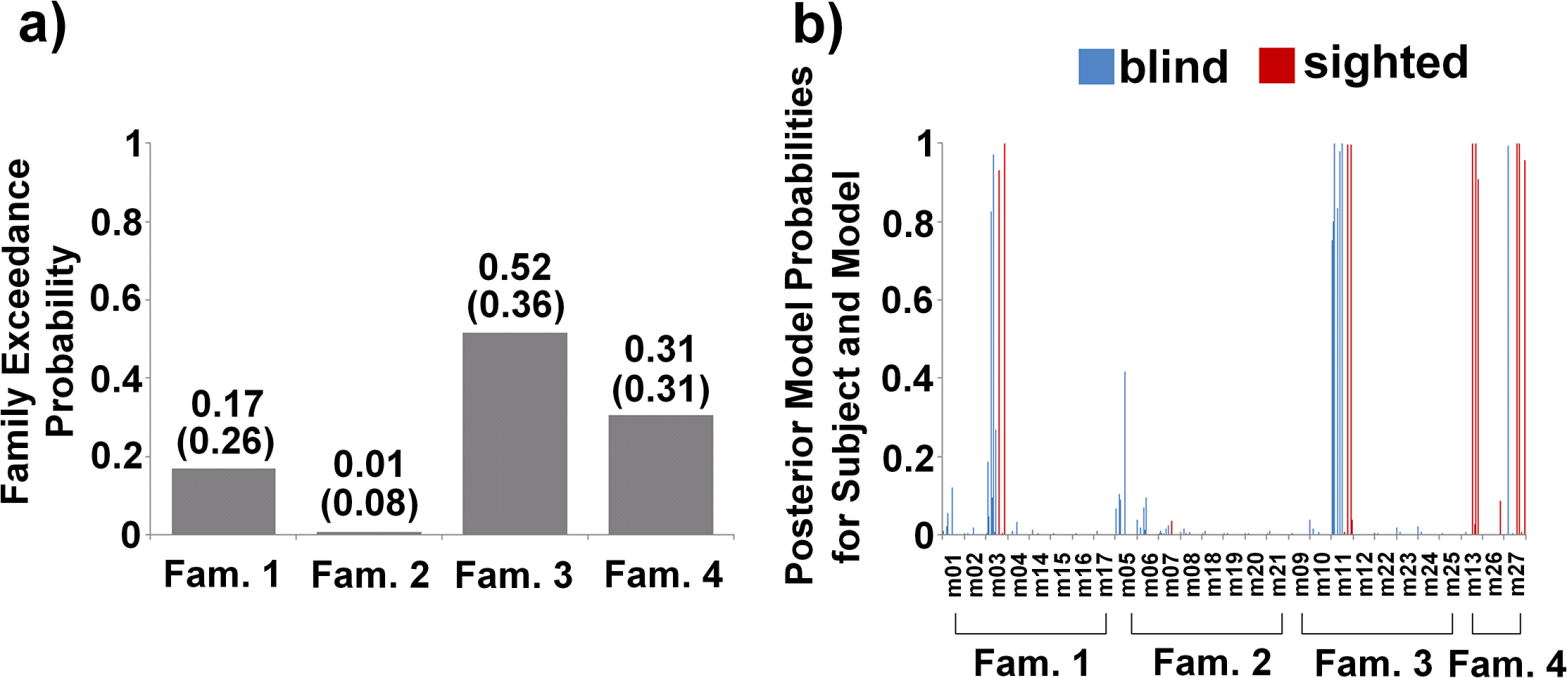
Bayesian model selection (BMS). (a) Exceedance probabilities and posterior expectations (in parentheses) resulting from the BMS procedure. The analysis did not reveal a clear winning family. But, with high confidence (total exceedance probability, *p* = 0.83), pre-SMA received either input from V1 (Fam. 3) or from A1, while Pv/V1 are not connected with the remaining areas (Fam. 4). (b) Posterior model probabilities for all subjects, assigned to the families (see also Table 2), indicating that blind subjects preferably chose Fam. 3, whereas Fam. 4 was much more likely for sighted individuals. More specifically, blind subjects showed high probability for the model m11, whereas sighted individuals primarily chose m13 or m27.

Fig 4A showed the posterior expectations and exceeding probabilities from the random-effects BMS procedure. The analysis did not yield a clear overall winning family. However, with high confidence (total exceedance probability, *p* = 0.83), pre-SMA received input either from V1 (Fam. 3, exceedance probability, *p* = 0.52) or from A1 in case Pv/V1 are not connected with the remaining areas (Fam. 4, exceedance probability, *p* = 0.31). Posterior model probabilities for subjects and models (Fig 4B, Table 2) revealed that blind subjects preferred Fam. 3 whereas Fam. 4 was much more likely for sighted individuals. More detailed, blind subjects showed high probability for a model with bidirectional connections between V1 and pre-SMA as well as Pv-A1/V1 (m11), whereas a few blind subjects chose a model which, additionally, included A1-pre-SMA coupling (m03, not winning family). The sighted group chose models with bidirectional (m13) or absent (m27) connectivity between A1 and pre-SMA, without any coupling from/to Pv/V1 (Fig 4B, Table 2).

**Table 2:** Posterior model probabilities for subjects and models resulting from the family-level inferences (BMS). Bold numbers indicate the most likely model (in Occam’s window) for each subject.

	Family 1								Family 2								Family 3								Family 4		
	m01	m02	m03	m04	m14	m15	m16	m17	m05	m06	m07	m08	m18	m19	m20	m21	m09	m10	m11	m12	m22	m23	m24	m25	m13	m26	m27
**Blind**																											
B01	0.01	0.00	0.00	0.00	0.00	0.00	0.00	0.00	0.07	0.04	0.00	0.01	0.01	0.01	0.01	0.00	0.00	0.04	**0.75**	0.00	0.00	0.02	0.02	0.00	0.00	0.00	0.00
B02	0.00	0.00	0.19	0.00	0.00	0.00	0.00	0.00	0.00	0.00	0.01	0.00	0.00	0.00	0.00	0.00	0.00	0.00	**0.80**	0.00	0.00	0.00	0.00	0.00	0.00	0.00	0.00
B03	0.00	0.00	0.00	0.00	0.00	0.00	0.00	0.00	0.00	0.00	0.00	0.00	0.00	0.00	0.00	0.00	0.00	0.00	**1.00**	0.00	0.00	0.00	0.00	0.00	0.00	0.00	0.00
B04	0.02	0.00	0.05	0.00	0.01	0.00	0.00	0.01	0.10	0.02	0.01	0.02	0.01	0.01	0.00	0.01	0.01	0.02	**0.68**	0.00	0.00	0.01	0.01	0.00	0.00	0.00	0.00
B05	0.00	0.00	0.00	0.00	0.00	0.00	0.00	0.00	0.00	0.00	0.00	0.00	0.00	0.00	0.00	0.00	0.00	0.00	0.00	0.00	0.00	0.00	0.00	0.00	0.01	0.00	**0.99**
B06	0.06	0.00	**0.83**	0.01	0.00	0.00	0.00	0.00	0.09	0.00	0.01	0.01	0.00	0.00	0.00	0.00	0.00	0.00	0.00	0.00	0.00	0.00	0.00	0.00	0.00	0.00	0.00
B07	0.00	0.00	0.09	0.00	0.00	0.00	0.00	0.00	0.00	0.07	0.00	0.00	0.00	0.00	0.00	0.00	0.00	0.00	**0.83**	0.00	0.00	0.00	0.00	0.00	0.00	0.00	0.00
B08	0.00	0.00	**0.97**	0.00	0.00	0.00	0.00	0.00	0.00	0.01	0.02	0.00	0.00	0.00	0.00	0.00	0.00	0.00	0.00	0.00	0.00	0.00	0.00	0.00	0.00	0.00	0.00
B09	0.00	0.00	0.01	0.00	0.00	0.00	0.00	0.00	0.00	0.00	0.00	0.00	0.00	0.00	0.00	0.00	0.00	0.01	**0.98**	0.00	0.00	0.00	0.00	0.00	0.00	0.00	0.00
B10	0.12	0.02	0.27	0.03	0.00	0.00	0.00	0.00	**0.42**	0.10	0.02	0.01	0.00	0.00	0.00	0.00	0.00	0.00	0.00	0.00	0.00	0.00	0.00	0.00	0.00	0.00	0.00
B11	0.00	0.00	0.00	0.00	0.00	0.00	0.00	0.00	0.00	0.00	0.00	0.00	0.00	0.00	0.00	0.00	0.00	0.00	**1.00**	0.00	0.00	0.00	0.00	0.00	0.00	0.00	0.00
**Sighted**																											
S01	0.00	0.00	0.00	0.00	0.00	0.00	0.00	0.00	0.00	0.00	0.00	0.00	0.00	0.00	0.00	0.00	0.00	0.00	0.00	0.00	0.00	0.00	0.00	0.00	**1.00**	0.00	0.00
S02	0.00	0.00	0.00	0.00	0.00	0.00	0.00	0.00	0.00	0.00	0.00	0.00	0.00	0.00	0.00	0.00	0.00	0.00	0.00	0.00	0.00	0.00	0.00	0.00	0.00	0.00	**1.00**
S03	0.00	0.00	0.00	0.00	0.00	0.00	0.00	0.00	0.00	0.00	0.00	0.00	0.00	0.00	0.00	0.00	0.00	0.00	0.00	0.00	0.00	0.00	0.00	0.00	**1.00**	0.00	0.00
S04	0.00	0.00	**1.00**	0.00	0.00	0.00	0.00	0.00	0.00	0.00	0.00	0.00	0.00	0.00	0.00	0.00	0.00	0.00	0.00	0.00	0.00	0.00	0.00	0.00	0.00	0.00	0.00
S05	0.00	0.00	0.00	0.00	0.00	0.00	0.00	0.00	0.00	0.00	0.00	0.00	0.00	0.00	0.00	0.00	0.00	0.00	**1.00**	0.00	0.00	0.00	0.00	0.00	0.00	0.00	0.00
S06	0.00	0.00	**1.00**	0.00	0.00	0.00	0.00	0.00	0.00	0.00	0.00	0.00	0.00	0.00	0.00	0.00	0.00	0.00	0.00	0.00	0.00	0.00	0.00	0.00	0.00	0.00	0.00
S07	0.00	0.00	0.00	0.00	0.00	0.00	0.00	0.00	0.00	0.00	0.00	0.00	0.00	0.00	0.00	0.00	0.00	0.00	0.00	0.00	0.00	0.00	0.00	0.00	0.00	0.00	**1.00**
S08	0.00	0.00	0.00	0.00	0.00	0.00	0.00	0.00	0.00	0.00	0.00	0.00	0.00	0.00	0.00	0.00	0.00	0.00	0.00	0.00	0.00	0.00	0.00	0.00	**0.91**	0.09	0.01
S09	0.00	0.00	**0.93**	0.00	0.00	0.00	0.00	0.00	0.00	0.00	0.04	0.00	0.00	0.00	0.00	0.00	0.00	0.00	0.01	0.00	0.00	0.00	0.00	0.00	0.03	0.00	0.00
S10	0.00	0.00	0.00	0.00	0.00	0.00	0.00	0.00	0.00	0.00	0.00	0.00	0.00	0.00	0.00	0.00	0.00	0.00	0.04	0.00	0.00	0.00	0.00	0.00	0.00	0.00	**0.96**
S11	0.00	0.00	0.00	0.00	0.00	0.00	0.00	0.00	0.00	0.00	0.00	0.00	0.00	0.00	0.00	0.00	0.00	0.00	**1.00**	0.00	0.00	0.00	0.00	0.00	0.00	0.00	0.00

### Model averaging (BMA)

Following up the family-level inferences using BMS, significance of driving input (A1/Pv) and intrinsic connections was tested by means of Bayesian model averaging (BMA) across all families. As proposed by Penny and colleagues [33], BMS combined with BMA is found to be useful when the posterior model density is not sharply peaked. The BMS (Fig 4) resulted in two families with highest evidence, which showed highest probability either for blind (Fam. 3) or sighted subjects (Fam. 4). Thus, the DCM parameters were tested for significance (parameter ≠ 0) separately for each subgroup.

**Fig 5:**
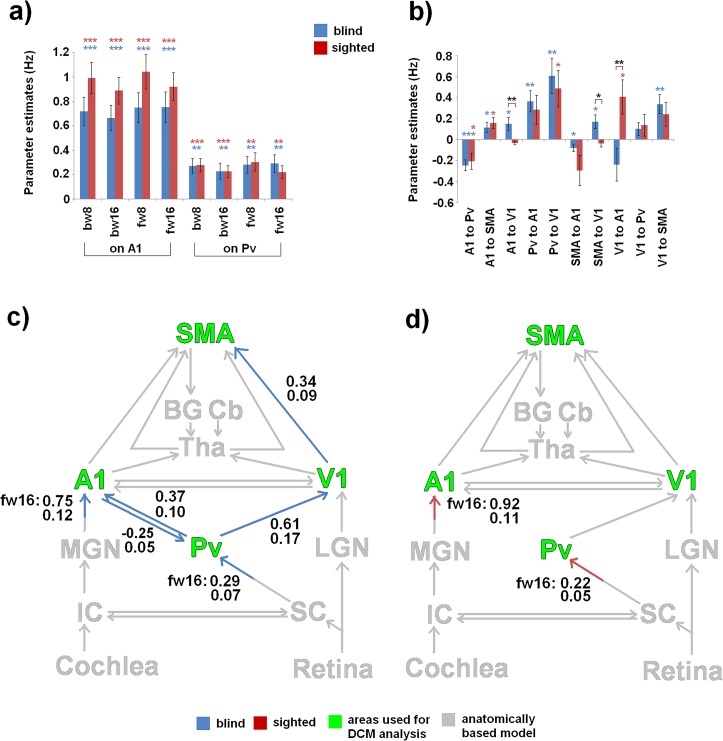
Bayesian model averaging (BMA). Individual DCM mean parameters of (a) driving input and (b) connection strength, tested for significance (parameter ≠ 0, one sample t test) separately for each subgroup (blind and sighted). Consistently for both subgroups and all speech conditions (bw8, bw16, fw8, fw16), driving input on A1 and Pv was highly significant. Significant values are represented by asterisks: *p* < 0.05 (*), *p* < 0.01 (**), *p* < 0.001 (***). Lower panels show Bonferroni Holm corrected data of blind (c) and sighted (d) individuals applied to the anatomically/functionally based network hypotheses (gray). Driving input is exemplified with the forward ultra-fast speech condition (fw16).

Consistently for both subgroups and all speech conditions (bw8, bw16, fw8, fw16), driving input on A1 and Pv was highly significant (A1: *p* < 0.001, Pv: *p* < 0.01; corr.) (Fig 5A, Table 3). Regarding the blind subgroup, connections from Pv to V1 (positive values, *p* < 0.01), from V1 to pre-SMA (positive values, *p* < 0.01), as well as from Pv to A1 (positive values, *p* < 0.01) and A1 to Pv (negative values, *p* < 0.001) were significant under Bonferroni Holm correction (Fig 5B and 5C, Table 3A). Coupling between A1 and pre-SMA as well as from pre-SMA/A1 to V1 were found to be significant, but not under Bonferroni Holm correction (Fig 5B and 5C, Table 3). Regarding the sighted subgroup, all connections between Pv, A1, V1, and pre-SMA were found to be non-significant under Bonferroni Holm correction (Fig 5B and 5D, Table 3).

**Table 3:** BMA results of the family-level BMS procedure (across blind and sighted). Parameter estimates (mean, standard error) of the driving input and intrinsic connectivity were calculated for each subgroup, blind and sighted, separately. Italic numbers indicate significance (*p* < 0.05), bold italic numbers indicate significance under Bonferroni Holm correction (connectivity: *p* < 0.005, driving input: *p* < 0.006).

**a) Intrinsic connectivity**										
	**A1 to Pv**	**A1 to SMA**	**A1 to V1**	**Pv to A1**	**Pv to V1**	**SMA to A1**	**SMA to V1**	**V1 to A1**	**V1 to Pv**	**V1 to SMA**
**Blind**										
Mean	***-0*.*25***	*0*.*12*	*0*.*15*	***0*.*37***	***0*.*61***	*-0*.*08*	*0*.*17*	-0.24	0.10	***0*.*34***
Standard error	***0*.*05***	*0*.*05*	*0*.*06*	***0*.*10***	***0*.*17***	*0*.*03*	*0*.*07*	0.16	0.06	***0*.*09***
**Sighted**										
Mean	*-0*.*21*	*0*.*16*	-0.03	0.28	*0*.*49*	-0.29	-0.04	*0*.*41*	0.14	0.24
Standard error	*0*.*08*	*0*.*05*	0.02	0.14	*0*.*18*	0.14	0.04	*0*.*16*	0.10	0.11
**b) Driving input**										
	**bw8 on A1**	**bw16 on A1**	**fw8 on A1**	**fw16 on A1**	**bw8 on Pv**	**bw16 on Pv**	**fw8 on Pv**	**fw16 on Pv**		
**Blind**										
Mean	***0*.*72***	***0*.*66***	***0*.*75***	***0*.*75***	***0*.*27***	***0*.*23***	***0*.*28***	***0*.*29***		
Standard error	***0*.*11***	***0*.*10***	***0*.*12***	***0*.*12***	***0*.*06***	***0*.*06***	***0*.*07***	***0*.*07***		
**Sighted**										
Mean	***0*.*99***	***0*.*89***	***1*.*04***	***0*.*92***	***0*.*28***	***0*.*23***	***0*.*30***	***0*.*22***		
Standard error	***0*.*13***	***0*.*11***	***0*.*14***	***0*.*11***	***0*.*05***	***0*.*05***	***0*.*07***	***0*.*05***		

**Fig 6:**
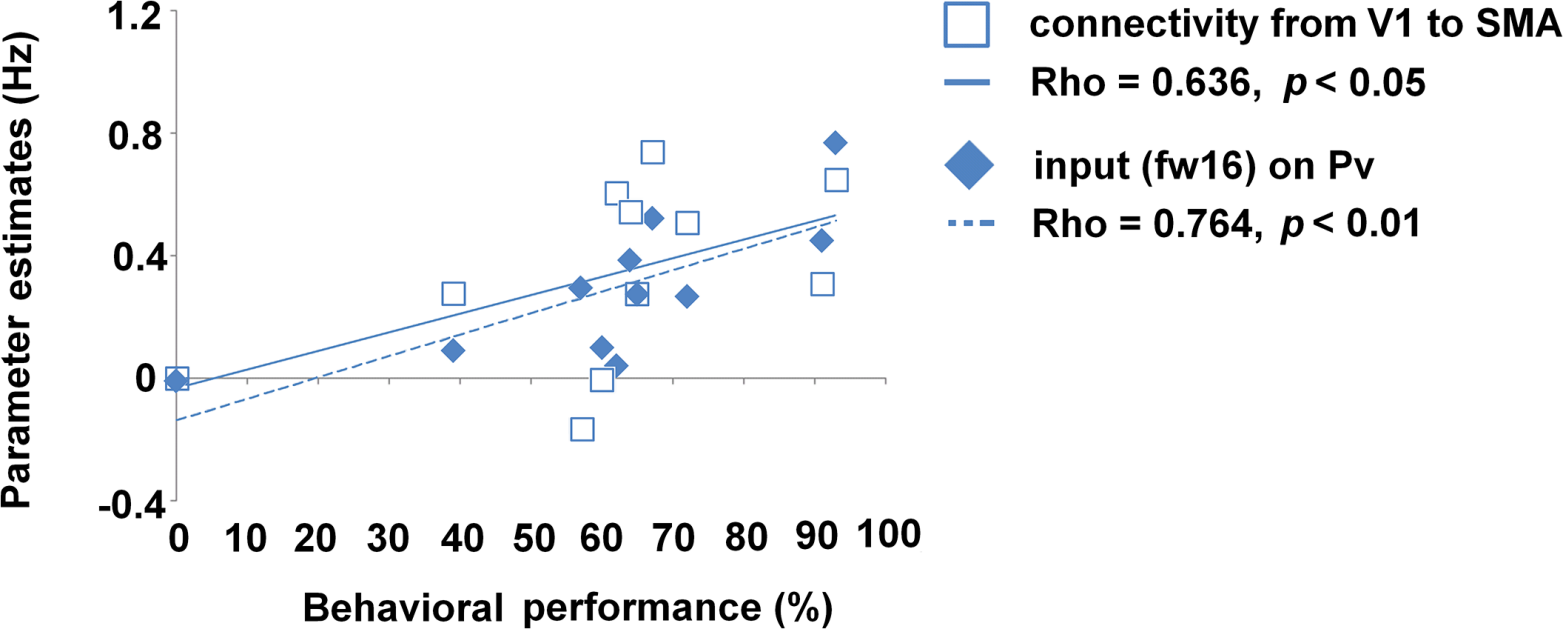
Correlation between DCM parameter and performance of ultra-fast speech comprehension. Connection strength of V1-pre-SMA (unfilled squares) and driving input on Pv (filled diamonds) plotted against individual behavioral performance of ultra-fast speech comprehension of the blind subgroup. Regression lines, correlation coefficients (Spearman Rho), and significance level were given.

A significant positive correlation between DCM parameters of the blind subgroup and behavioral performance of ultra-fast speech comprehension was expected and could be found regarding (i) connectivity from V1 to pre-SMA (two-tailed Spearman test; *ρ* = 0.636, *p* < 0.05, uncorr., Fig 6, S2 Table) as well as (ii) driving input on Pv during the ultra-fast speech condition (two-tailed Spearman test; fw16: *ρ* = 0.764, *p* < 0.01, corr., Fig 6, S2 Table). Additionally, not significant, but tentative, parameters of Pv-V1 connection were found to be correlated with performance of ultra-fast speech comprehension of blind individuals (two-tailed Spearman test; *ρ* = 0.582, *p* = 0.06, S2 Table). Regarding correlations between DCM parameters and the age of blindness onset neither intrinsic connections nor driving input reached significance under Bonferroni Holm correction (S2 Table). As concerns sighted participants no correlation at all could be found (S2 Table).

Regarding intrinsic connections, no significant differences under Bonferroni Holm correction were found between blind and sighted subjects (S3 Table). However, V1-A1 (*p* < 0.01) as well as pre-SMA-V1 (*p* < 0.05) coupling seems to differ (significant, but uncorr.) in the way that blind and sighted subjects showed an inverse pattern: positive values (i) from V1 to A1 in sighted subjects and (ii) from A1 to V1 in blind individuals (Fig 5B, S3 Table).

## Discussion

In this study, the skill of ultra-fast speech comprehension of blind individuals was investigated using dynamic causal modeling in the context of a speech perception fMRI paradigm. A hypothesis of visual cortex involvement in this ability was investigated by testing functional connectivity within a hypothesized network comprising right Pv, A1, V1, and left pre-SMA. Using BMS, a family of models coupling V1 and pre-SMA (Fam. 3) as well as Pv and V1/A1 was identified as the best family in the blind subject group. By contrast, in sighted controls connectivity from/to Pv/V1 did not play a significant role during speech perception (Fam. 4). The BMA procedure showed significant coupling from Pv to V1/A1 as well as from visual cortex to pre-SMA in the blind subgroup. By contrast, sighted participants did not show any significant intrinsic connection under Bonferroni Holm correction, although driving input on Pv/A1 was significant with respect to all conditions (fw8, bw8, bw16, fw16). As expected, in blind individuals the forward connection from V1 to pre-SMA as well as driving input on Pv during forward ultra-fast speech were found to be significantly correlated with the capability of ultra-fast speech comprehension. Furthermore, blind and sighted subjects did not differ significantly (under Bonferroni Holm correction) with respect to intrinsic connectivity and driving input. Although none of the connections from/to Pv/V1 reached significance in sighted subjects, it is presumed that these connections are principally available, but not for the present unimodal auditory task of listening to speech. Furthermore, A1-V1 coupling tended to show reverse values in sighted as compared to blind subjects (blind: A1-V1 positive, sighted: V1-A1 positive values). Thus, in blind subjects V1 seems to receive cortico-cortical auditory input, whereas sighted subjects rather facilitate influences from V1 to A1.

### Secondary visual pathway

Blind subjects perceiving ultra-fast speech may use an alternative prosodic channel via an afferent audiovisual pathway including superior colliculus (SC), pulvinar (Pv), and right visual cortex [10, 12]. In sighted subjects, these pathways contribute to auditory-driven gating and timing mechanisms for visual object recognition and/or are involved in visual mechanisms of spatial recalibration for auditory events [12]. Due to functional reorganization, blind subjects may use these auditory afferent pathways toward the visual system during ultra-fast speech perception, providing the visual system with an auditory temporal event structure.

The “classical” visual pathway is that from the retina via LGN to V1, but several authors postulate a second visual pathway extending from retina via the superficial layers of the SC to Pv of the thalamus (tecto-pulvinar) and from there to V1 (pulvino-cortical) [37]. Considering the location of the Pv nuclei involved in tecto-pulvino-cortical interactions, neurochemical studies [38–40] as well as electrophysiological recording [41–44] indicated the inferior lateral part of the Pv posterior to LGN functioning as relay station. Furthermore, using micro-stimulation of SC, Pv, and the visual motion area (in monkeys), Berman and Wurtz [45] found clusters of neurons in the Pv that receive input from SC along with neurons that project or receive information to/from the visual motion area. They suggested that secondary visual pathway pass through the thalamic pulvinar nucleus and project to multiple regions of visual cortex. Although these pathways have been reported to target higher rather than primary visual areas [45–47], a diffusion tensor imaging tractography study indicates also connectivity from Pv to early visual cortex [48]. Furthermore, Petersen and colleagues [43] found in inferior lateral Pv of rhesus monkeys attention-modulated neurons with short response latencies. Considering these previous findings, the input region of the present DCM analysis, being exactly located within inferior lateral Pv, most likely, belongs to the secondary visual pathway. As concerns the winning family as well as significant connectivity pattern of the blind subgroup in the present DCM analysis, Pv showed positive coupling with V1 as well as with A1, whereas connection from A1 to Pv was found to be negative. This seems to be in line with functional-anatomical suggestions (see above): the present Pv region appears to be part of the visual stream, but also reflects an interface to the auditory pathway synchronizing auditory and visual cortices [16]. Furthermore, regarding the reorganized visual system in blind subjects, DCM revealed significant forward connection from Pv to V1 indicating a bottom-up rather than top-down information flow toward the visual system. Generally, cortico-pulvinar output was found in macaque monkeys to arise from layer 5 of V1 [49–50], whereas pulvino-cortical projections terminated in superficial layers of V1, mainly layer 1 [51–53]. Note that Pv was, additionally, linked to higher level visual areas, e.g., V2 and V4 ([54] for more details). Although, first, differential laminar patterns cannot be assessed in DCM analysis and, second, forward versus backward connectivity should be distinguished from the cognitive aspects of bottom-up versus top-down processing [55], the present connection from Pv to V1 might represent an early stage of speech processing in the present speech perception paradigm. Considering the model about mechanisms of ultra-fast speech comprehension proposed by Hertrich and colleagues [12], right V1, in addition to A1, has been assumed to receive early speech information, i.e. timing of syllable onsets, triggering phonological processing in the frontal cortex. Thus, forward connection from Pv to V1 might reflect the pathway on which these early prosodic cues come into V1. Interestingly, blind individuals did not show any significant coupling between A1 and V1, although this might be hypothesized on the basis of anatomical considerations [23–25]. Thus, in the present experiment functional audio-visual coupling in blind subjects seems to occur at the subcortical (Pv) rather than cortical (A1-V1) level. Regarding a recent DCM study [56], the authors documented that cortico-cortical connection strengths between primary auditory and visual cortex were stronger in blind as compared to sighted subjects, whereas thalamo-cortical connectivity between the MGN/LGN and primary visual/auditory system did not differ between both subgroups. Subsequently, they concluded that in blind listeners V1 receives auditory information from the primary auditory cortex via cortico-cortical connections. This might be reflected in the present findings of cortico-cortical A1-V1 (significant, but without Bonferroni Holm correction) coupling, in addition to Pv-V1 connection. Wong and Bhattacharjee [57] comment on the findings of Klinge and colleagues [56] the controversial issue of how the primary visual cortex receives auditory information in blind listeners–via cortico-cortical or thalamo-cortical connections. Thus, discussions about cortico-cortical or thalamo-cortical connections of V1 raise a wide range of questions aiming at the function of V1 during non-visual tasks: (i) bottom-up (thalamo-cortical) versus top-down (cortico-cortical) processes, (ii) directionality of cortico-cortical A1-V1 coupling during auditory perception, and (iii) effects of the experimental task on the strength of thalamo-cortical versus cortico-cortical connectivity? Klinge and colleagues [56] reported bidirectional coupling between A1 and V1, whereas only A1 to V1 coupling reached significance under Bonferroni Holm correction and values of connectivity parameters were positive. Accordingly, although not significant under Bonferroni Holm correction, the present study revealed positive values of connectivity parameters only from A1 to V1, whereas negative V1-A1 coupling was not significant in blind subjects. These differences might result from distinct tasks used in the studies: Klinge and colleagues [56] applied meaningless bisyllabic pseudowords while probands were asked to rate emotional prosody or vowel quality. This task did not require a superordinate linguistic prosody for the tracking of continuous speech as in the present ultra-fast sentence materials. Thus, information flow (positive connectivity values) from V1 to A1 –speculatively interpreted as “feedback”–may not inhibit the process during such a single item task. By contrast, processing ultra-fast spoken sentences may exceed the prosodic resolution capacity of A1 and, therefore, avoiding “feedback” and facilitating bottom up processing in V1, inhibitory connectivity from V1 to A1 (negative values of connectivity parameters) as well as facilitating coupling from A1 to V1 (positive values of connectivity parameters) seems plausible. However, a consensus on the interpretation and neurophysiological function of forward/backward coupling and negative/positive values of connectivity parameters has not been reached yet in literature.

### From sensory cortices toward the frontal lobe

Synchronization of the left-lateralized phonological system with the incoming acoustic signal via a right-hemispheric prosodic trigger mechanism appears to represent an important prerequisite for continuous speech perception under time-critical conditions. Frontal cortex, particularly, SMA, seems to be involved in the coordination of phonological encoding with prosodic timing via subcortical structures, i.e., BG, Tha, and Cb (see [28]). Based on considerations such as the “dynamic dual pathway model” [58] and the "asymmetric sampling in time hypothesis [59], speech perception comprises two partially independent data streams, one representing left-hemisphere dominant phonological and syntactic processing while the other provides a prosodic signal that, in sighted subjects, is predominantly represented in the right-hemispheric auditory system. Moreover, visual cortex activity in blind humans was found to reflect language processing which, additionally, seem to be lateralized at the occipital level according to its prominent features, i.e., left-side in case of phonological processing [60–61] or right side regarding prosodic focusing [10, 13]. Regarding the temporal coordination between the two subsystems, SMA and pre-SMA as components of a subcortical/cortical timing network seem to play an important role [28, 62].

Considering ultra-fast speech perception, pre-SMA was found to be more activated in high-performing (blind) than in naïve subjects [10]. In line with the syllabic timing hypothesis with respect to V1 recruitment (see above), high-performing blind subjects showed functional connectivity between V1 and pre-SMA–presumably via indirect subcortical nodes not defined in the present model space. Coupling between A1 and pre-SMA was also observed in the blind as well as sighted subjects, but failed significance after Bonferroni Holm correction. However, previous fMRI findings [13] showed considerable variation across individuals in hemodynamic activation of pre-SMA during moderately fast speech perception, suggesting that connectivity between A1 and pre-SMA does not seem to be a prerequisite to speech comprehension per se, but might only be necessary in case of higher processing load or task difficulty (see, e.g., [63]). Additional evidence for the task relevance of V1-pre-SMA connectivity in blind subjects was provided by the presence of a significant positive correlation between connection strength and behavioral performance of understanding ultra-fast speech. Thus, switching from A1-pre-SMA to V1-pre-SMA coupling might indicate a change in strategy used to accelerate speech perception beyond the event recording capacity of the auditory system at ca. 16 Hz where event rates tend to melt into a tonal percept [64].

To summarize, increased connectivity from V1 to pre-SMA with increasing performance of ultra-fast speech comprehension in blind individuals confirm the importance of a second sensory channel (V1), in addition to the auditory system (A1) in order to forward syllabic information from the sensory level to the frontal speech processing network.

## Conclusions

Two pathways of the central-visual system (right V1) seem to support ultra-fast speech comprehension in blind subjects: first, early subcortical representations of the speech signal, e.g., segmental boundaries (syllable onsets), are conveyed via the Pv to right-hemisphere V1 (= secondary visual pathway); second, coupling from right V1 to contralateral pre-SMA triggers the encoding process toward inner speech representations and verbal working memory. Thus, V1 might facilitate the “parsing” of the ultra-fast speech signal in syllable-sized units.

## Supporting Information

S1 Table Coordinates of each peak from each participant (blind = B, sighted = S) as well as the behavioral performance of understanding ultra-fast speech were listed. If a participant did not show significant hemodynamic responses (during the “forward ultra-fast speech” condition) in a search region (primary auditory cortex = A1, primary visual cortex = V1, pulvinar = Pv, anterior part of supplementary motor area (pre-SMA)), the group coordinate (bold values) resulting from the previous fMRI study [10] was used determining the volume of interest. Coordinates are denoted in Montreal Neurological Institute (MNI) space.
(DOCX)

S2 Table Correlation (Spearman Rho, two-tailed) between DCM parameters and the behavioral performance of ultra-fast speech as well as the age of blindness onset. Italic numbers indicate significance (*p* < 0.05), bold italic numbers indicate significance under Bonferroni Holm correction (only applied to driving input: *p* < 0.006).
(DOCX)

S3 Table Differences between blind and sighted individuals within DCM parameters (one way ANOVA). Italic numbers indicate significance (*p* < 0.05), bold italic numbers indicate significance under Bonferroni Holm correction (connectivity: *p* < 0.005, driving input: *p* < 0.006).
(DOCX)
