# Supplementary material for: Network Modeling for Functional Magnetic Resonance Imaging (fMRI) Signals during Ultra-Fast Speech Comprehension in Late-Blind Listeners
Source: PLoS One. 2015 Jul 6;10(7):e0132196. doi: 10.1371/journal.pone.0132196 (PMC4492787; doi:10.1371/journal.pone.0132196)
Supplement: S1 Table — If a participant did not show significant hemodynamic responses (during the “forward ultra-fast speech” condition) in a search region (primary auditory cortex = A1, primary visual cortex = V1, pulvinar = Pv, anterior part of supplementary motor area (pre-SMA)), the group coordinate (bold values) resulting from the previous fMRI study [10] was used determining the volume of interest. Coordinates are denoted in Montreal Neurological Institute (MNI) space. (DOCX) [file pone.0132196.s001.docx]

**S1 Table.** Coordinates of each peak from each participant (blind = B, sighted = S) as well as the behavioral performance of understanding ultra-fast speech were listed. If a participant did not show significant hemodynamic responses (during the “forward ultra-fast speech” condition) in a search region (primary auditory cortex = A1, primary visual cortex = V1, pulvinar = Pv, anterior part of supplementary motor area (pre-SMA)), the group coordinate (bold values) resulting from the previous fMRI study [10] was used determining the volume of interest. Coordinates are denoted in Montreal Neurological Institute (MNI) space.

| **Subjects** | **Performance of ultra-fast speech comprehension (%)** | **Coordinates (x, y, z)** | | | | | | | | | | | |
| --- | --- | --- | --- | --- | --- | --- | --- | --- | --- | --- | --- | --- | --- |
|  |  | **A1** | | | **V1** | | | **Pv** | | | **Pre-SMA** | | |
|  |  | **x** | **y** | **z** | **x** | **y** | **z** | **x** | **y** | **z** | **x** | **y** | **z** |
| B01 | 93 | 57 | -9 | 3 | 21 | -99 | -6 | 17 | -27 | -6 | -3 | 12 | 60 |
| B02 | 91 | 57 | -15 | 3 | 21 | -102 | 9 | 18 | -30 | -3 | -9 | 12 | 54 |
| B03 | 72 | 57 | -9 | 3 | 21 | -102 | 12 | 18 | -30 | -3 | -6 | 9 | 54 |
| B04 | 67 | 57 | -6 | 3 | 12 | -102 | 9 | 18 | -27 | -6 | -3 | 12 | 54 |
| B05 | 65 | 54 | -9 | -3 | 15 | -96 | 3 | 18 | -30 | -3 | 15 | -96 | 3 |
| B06 | 64 | 54 | -15 | 9 | 12 | -99 | 12 | 18 | -27 | -6 | 0 | 3 | 60 |
| B07 | 62 | 51 | -18 | -3 | 21 | -102 | 0 | **18** | **-30** | **-6** | 0 | 6 | 63 |
| B08 | 60 | 51 | -18 | 3 | 21 | -102 | 12 | **18** | **-30** | **-6** | 0 | 12 | 60 |
| B09 | 57 | 57 | -15 | -3 | 21 | .96 | 6 | **18** | **-30** | **-6** | -6 | 6 | 54 |
| B10 | 39 | 57 | -15 | 0 | 12 | -102 | 0 | **18** | **-30** | **-6** | **-6** | **9** | **60** |
| B11 | 0 | 57 | -6 | 3 | **15** | **-102** | **6** | **18** | **-30** | **-6** | **-6** | **9** | **60** |
| S01 | 16 | 57 | -18 | 3 | **15** | **-102** | **6** | 3 | -30 | -6 | 0 | 6 | 57 |
| S02 | 16 | 57 | -12 | 0 | **15** | **-102** | **6** | **18** | **-30** | **-6** | **-6** | **9** | **60** |
| S03 | 11 | 54 | -15 | 0 | **15** | **-102** | **6** | **18** | **-30** | **-6** | 0 | 9 | 60 |
| S04 | 6 | 57 | -9 | 0 | **15** | **-102** | **6** | **18** | **-30** | **-6** | 0 | 12 | 60 |
| S05 | 5 | 57 | -9 | 0 | **15** | **-102** | **6** | **18** | **-30** | **-6** | **-6** | **9** | **60** |
| S06 | 4 | 54 | -6 | 3 | **15** | **-102** | **6** | **18** | **-30** | **-6** | 0 | 3 | 60 |
| S07 | 9 | 57 | -12 | 3 | **15** | **-102** | **6** | **18** | **-30** | **-6** | **-6** | **9** | **60** |
| S08 | 7 | 57 | -12 | 6 | **15** | **-102** | **6** | 15 | -30 | -6 | **-6** | **9** | **60** |
| S09 | 16 | 57 | -12 | -3 | **15** | **-102** | **6** | 15 | -30 | -6 | 0 | 12 | 57 |
| S10 | 6 | 54 | -15 | 9 | **15** | **-102** | **6** | **18** | **-30** | **-6** | 0 | 12 | 60 |
| S11 | 8 | 57 | -12 | 6 | **15** | **-102** | **6** | **18** | **-30** | **-6** | **-6** | **9** | **60** |
