# Supplementary material for: Network Modeling for Functional Magnetic Resonance Imaging (fMRI) Signals during Ultra-Fast Speech Comprehension in Late-Blind Listeners
Source: PLoS One. 2015 Jul 6;10(7):e0132196. doi: 10.1371/journal.pone.0132196 (PMC4492787; doi:10.1371/journal.pone.0132196)
Supplement: S2 Table — Italic numbers indicate significance (p < 0.05), bold italic numbers indicate significance under Bonferroni Holm correction (only applied to driving input: p < 0.006). (DOCX) [file pone.0132196.s002.docx]

**S2 Table.** Correlation (Spearman Rho, two-tailed) between DCM parameters and the behavioral performance of ultra-fast speech as well as the age of blindness onset. Italic numbers indicate significance (*p* < 0.05), bold italic numbers indicate significance under Bonferroni Holm correction (only applied to driving input: *p* < 0.006).

| 1. **Intrinsic connectivity** | | | | | | | | | | |
| --- | --- | --- | --- | --- | --- | --- | --- | --- | --- | --- |
|  | **A1 to Pv** | **A1 to SMA** | **A1 to V1** | **Pv to A1** | **Pv to V1** | **SMA to A1** | **SMA to V1** | **V1 to A1** | **V1 to Pv** | **V1 to SMA** |
| **Blind** |  |  |  |  |  |  |  |  |  |  |
| Performance |  |  |  |  |  |  |  |  |  |  |
| *Rho* | -.446 | .079 | -.409 | .282 | .582 | -.163 | -.345 | -.009 | .164 | *.636* |
| *p-value* | .170 | .817 | .212 | .401 | .060 | .632 | .298 | .979 | .631 | *.035* |
| Onset of blindness |  |  |  |  |  |  |  |  |  |  |
| *Rho* | .431 | .275 | .241 | -.306 | -.211 | -.376 | .211 | -.211 | -.040 | .286 |
| *p-value* | .185 | .413 | .476 | .360 | .534 | .254 | .534 | .534 | .907 | .394 |
| **Sighted** |  |  |  |  |  |  |  |  |  |  |
| Performance |  |  |  |  |  |  |  |  |  |  |
| *Rho* | .506 | .100 | -.173 | .146 | .014 | .455 | -.282 | .269 | .241 | -.305 |
| *p-value* | .113 | .769 | .611 | .669 | .968 | .160 | .400 | .424 | .474 | .361 |
| 1. **Driving input** | | | | | | | | | | |
|  | **bw8 on A1** | **bw16 on A1** | **fw8 on A1** | **fw16 on A1** | **bw8 on Pv** | **bw16 on Pv** | **fw8 on Pv** | **fw16 on Pv** |  |  |
| **Blind** |  |  |  |  |  |  |  |  |  |  |
| Performance |  |  |  |  |  |  |  |  |  |  |
| *Rho* | .391 | .427 | .309 | .536 | *.645* | .536 | .591 | ***.764*** |  |  |
| *p-value* | .235 | .190 | .355 | .089 | *.032* | .089 | .056 | ***.006*** |  |  |
| Onset of blindness |  |  |  |  |  |  |  |  |  |  |
| *Rho* | .575 | *-.633* | -.569 | -.569 | *-.642* | -.506 | *-.706* | *-.661* |  |  |
| *p-value* | .109 | *.036* | .067 | .067 | *.033* | .113 | *.015* | *.027* |  |  |
| **Sighted** |  |  |  |  |  |  |  |  |  |  |
| Performance |  |  |  |  |  |  |  |  |  |  |
| *Rho* | .575 | .547 | .593 | .575 | -.166 | -.262 | -.083 | -.198 |  |  |
| *p-value* | .064 | .082 | .054 | .064 | .627 | .436 | .809 | .560 |  |  |
