# Supplementary material for: Network Modeling for Functional Magnetic Resonance Imaging (fMRI) Signals during Ultra-Fast Speech Comprehension in Late-Blind Listeners
Source: PLoS One. 2015 Jul 6;10(7):e0132196. doi: 10.1371/journal.pone.0132196 (PMC4492787; doi:10.1371/journal.pone.0132196)
Supplement: S3 Table — Italic numbers indicate significance (p < 0.05), bold italic numbers indicate significance under Bonferroni Holm correction (connectivity: p < 0.005, driving input: p < 0.006). (DOCX) [file pone.0132196.s003.docx]

**S3 Table.** Differences between blind and sighted individuals within DCM parameters (one way ANOVA). Italic numbers indicate significance (*p* < 0.05), bold italic numbers indicate significance under Bonferroni Holm correction (connectivity: *p* < 0.005, driving input: *p* < 0.006).

| 1. **Intrinsic connectivity** | | | | | | | | | | |
| --- | --- | --- | --- | --- | --- | --- | --- | --- | --- | --- |
|  | **A1 to Pv** | **A1 to SMA** | **A1 to V1** | **Pv to A1** | **Pv to V1** | **SMA to A1** | **SMA to V1** | **V1 to A1** | **V1 to Pv** | **V1 to SMA** |
| *F* | .179 | .341 | *8.144* | .223 | .246 | 2.106 | *7.415* | *8.157* | .093 | .449 |
| *p-value* | .677 | .566 | *.010* | .642 | .625 | .162 | *.013* | *.010* | .763 | .511 |
| 1. **Driving input** | | | | | | | | | | |
|  | **bw8 on A1** | **bw16 on A1** | **fw8 on A1** | **fw16 on A1** | **bw8 on Pv** | **bw16 on Pv** | **fw8 on Pv** | **fw16 on Pv** |  |  |
| *F* | 2.527 | 2.241 | 2.484 | 1.033 | .006 | .001 | .051 | .613 |  |  |
| *p-value* | .128 | .150 | .131 | .322 | .037 | .972 | .824 | .443 |  |  |
